# Optimization of Somatic Embryogenesis and Plant Regeneration in Areca Palm (*Areca catechu* L.)

**DOI:** 10.3390/plants15142151

**Published:** 2026-07-13

**Authors:** Qikai Zhang, Qiongxiang Li, Yu Li, Chao Ouyang, Jixin Zou, Dongdong Li

**Affiliations:** 1 National Key Laboratory for Tropical Crop Breeding, College of Tropical Agriculture and Forestry, Hainan University, Sanya 572025, China; zhangqikai@hainanu.edu.cn (Q.Z.); liqiongxiang0407@163.com (Q.L.); liyutz2001@163.com (Y.L.); 2 Coconut Research Institute of Chinese Academy of Tropical Agricultural Sciences (CATAS), Wenchang 571339, China; oycsuper@163.com (C.O.); zourich@163.com (J.Z.)

**Keywords:** areca catechu, somatic embryogenesis, cell totipotency, embryogenic competence, cytokinin-induced auxin synthesis

## Abstract

Somatic embryogenesis (SE)—the reprogramming of somatic cells to follow an embryogenic pathway—remains inefficient in the tropical monocot areca palm (*Areca catechu* L.) due to poorly understood constraints on cell fate transition. Using single-factor and orthogonal experiments, we investigated how plant growth regulators (PGRs), nitrogen source, and carbon supply regulate the acquisition of embryogenic competence. The optimal condition (Y3 medium + 6-BA + TDZ + 30 g/L sucrose) yielded a somatic embryo induction rate of 48.62%, more than six-fold higher than that of the unoptimized control. Cytologically, embryogenic callus consisted of small, densely cytoplasmic cells with large, centrally positioned nuclei with decondensed chromatin (i.e., loosely packed, transcriptionally active chromatin) and minimal vacuolation—hallmarks of meristematic, embryogenic-competent cells—whereas non-embryogenic callus contained large, highly vacuolated irregular cells. Histological tracking revealed that areca somatic embryos faithfully recapitulate the zygotic developmental program, including bipolarity establishment and procambium differentiation. Mechanistically, we show that the 6-BA + TDZ combination likely acts by inducing endogenous auxin synthesis; moderate ammonium supply (Y3 medium, NO_3_^−^/NH_4_^+^ ratio 3.6:1) minimizes browning; and 30 g/L sucrose provides optimal energy supply and osmotic regulation. This optimized SE system establishes a tractable model for studying somatic cell reprogramming in a woody monocot.

## 1. Introduction

Areca palm (*Areca catechu* L.), a perennial evergreen arborescent monocot of the family Arecaceae, is an economically important cash crop widely cultivated in tropical and subtropical regions, particularly in Hainan Province, China, where it serves as a pillar industry for local farmers [1,2]. However, the conventional propagation of areca palm relies almost exclusively on seed propagation. Due to its cross-pollination nature and long juvenile phase, seed-derived seedlings exhibit severe trait segregation, making it difficult to maintain superior agronomic characteristics across generations [2,3]. Moreover, vegetative propagation methods such as cutting or grafting are not applicable to this single-trunk, non-branching species [2]. Consequently, there is a pressing need for an efficient and reliable clonal propagation system to accelerate the deployment of elite germplasm and to support the sustainable development of the areca industry.

Somatic embryogenesis (SE)—the process by which somatic cells undergo dedifferentiation and reprogramming to follow an embryogenic developmental pathway, ultimately giving rise to complete plants without gamete fusion—represents the most complete expression of plant cell totipotency [4,5]. Compared with organogenesis, SE offers several distinctive advantages: it bypasses the intermediate rooting/shooting steps, produces bipolar structures containing both apical and basal meristems in a single event, and enables high-frequency clonal propagation with theoretically uniform genetic backgrounds [6,7]. Since the first report of SE in carrot [8], this technology has been successfully established in numerous plant species, including many tropical woody crops such as oil palm, coconut, and date palm [9,10].

In the Arecaceae family, substantial progress has been made in SE systems. Oil palm (*Elaeis guineensis*) was among the first palm species to achieve large-scale clonal propagation via SE, although long-term culture-induced somaclonal variation (the “mantled” fruit abnormality) remains a challenge [11]. Coconut (*Cocos nucifera*) and date palm (*Phoenix dactylifera*) have also been successfully regenerated through SE using various explants including immature inflorescences and zygotic embryos [12,13]. For areca palm, pioneering work by Karun and colleagues demonstrated that SE could be induced from zygotic embryos, leaf explants, and immature inflorescences [14,15]. Subsequently, Wang et al. extended the explant sources to leaf, root, and stem segments and described the morphological progression of areca somatic embryos from globular to cotyledonary stages [16]. Despite these efforts, the areca SE system remains far from optimal: somatic embryo induction rates are generally low (typically below 20%); browning and necrosis are severe, and the system has not yet achieved industrial-scale application [9,16].

The low efficiency of areca SE is likely attributable to multiple factors, including inappropriate plant growth regulator (PGR) regimes, suboptimal basal medium composition, and insufficient understanding of the cellular and developmental constraints that limit somatic cell reprogramming. Exogenous PGRs added to the culture medium act as chemical cues that modulate endogenous hormone synthesis, gradient formation, and chromatin remodeling [17,18]. In palms, 2,4-dichlorophenoxyacetic acid (2,4-D) has been widely used for embryogenic callus induction, but its continuous presence often inhibits embryo development and promotes browning [19]. Cytokinins such as 6-benzyladenine (6-BA) and thidiazuron (TDZ) are known to promote the differentiation of embryogenic cells and to improve embryo quality in recalcitrant species [20]. It has been proposed that TDZ can act as an inducer of endogenous auxin and gibberellin synthesis, rather than serving as a direct auxin source. In addition, basal medium composition—<font color = “red”>especially the overall nutrient balance, including N:P:K ratios and trace element composition profoundly affects cellular metabolism, stress responses, and the progression of embryogenic development [21]. For areca palm, systematic optimization integrating PGRs, basal media, and carbon/nitrogen sources has not yet been reported.

Furthermore, a mechanistic understanding of areca SE requires cellular-level characterization of the transition from non-embryogenic to embryogenic states. This transition, which can be viewed as an epigenetic reprogramming event, involves profound changes in chromatin organization—including alterations in chromatin entropy, packing density, and histone modification patterns. Local endogenous hormone production may act as an upstream factor triggering these chromatin changes. Embryogenic cells typically exhibit a characteristic cytology: small isodiametric shape, dense cytoplasm, a large, centrally located nucleus with deeply stained and decondensed chromatin (reflecting high transcriptional activity), a high nucleus-to-cytoplasm ratio, and minimal vacuolation [4,22]. These features reflect active cell division and the retention of embryogenic potential. Non-embryogenic cells, in contrast, are large, highly vacuolated, and irregularly shaped, with little regenerative capacity. Histological tracking of developmental stages—from globular to cotyledonary embryos—can reveal whether somatic embryos faithfully recapitulate the ontogenetic program of zygotic embryos, including the establishment of bipolarity, procambium differentiation, and apical meristem formation. Such cytological evidence is essential for validating the authenticity and quality of SE systems but has been only superficially addressed in areca palm.

In this study, we therefore aimed to (i) systematically optimize the key factors influencing areca SE, including PGR combinations, basal media, and carbon/nitrogen sources, using single-factor and orthogonal experimental designs; (ii) characterize the cytological differences between embryogenic and non-embryogenic callus, and track the histological changes across successive stages of somatic embryo development; and (iii) establish an efficient and reliable SE protocol for areca palm that can serve both as a platform for clonal propagation and as a model system for studying cell fate reprogramming in a tropical woody monocot. In this paper “somatic embryo differentiation” is defined as the transition from proliferating embryogenic callus cells to distinct, polarized, globular-stage somatic embryos, representing the initial morphological manifestation of cell fate commitment to the embryogenic pathway.

## 2. Results

### 2.1. Optimization of PGR Combinations for Somatic Embryo Differentiation

To identify the most effective PGR regime for inducing somatic embryo differentiation, nine different PGR combinations were tested using the Y3 basal medium. After 60 days of dark culture, all treatments successfully induced somatic embryos, with induction rates significantly higher than those in the unoptimized control (2 mg/L 2,4-D + 0.5 mg/L Kin, induction rate 7.76 ± 2.87%) (Table 1). Among the nine treatments, the combination of 6-BA and TDZ (Treatment 8) yielded the highest somatic embryo induction rate (27.40 ± 3.60%), which was more than 3.5 times that of the control. This treatment also maintained a relatively low browning mortality rate (9.53 ± 1.61%), comparable to the control level. In contrast, treatments containing 2,4-D or high concentrations of auxins (e.g., Treatment 7 with Pic, Treatment 9 with 2,4-D) showed substantially higher browning mortality rates (>22%), indicating that high concentrations of these synthetic compounds may induce cellular stress in non-competent cells, leading to browning and necrosis. Notably, while controlled stress can promote SE induction in competent cells, excessive stress in non-competent cells triggers cell death.

**Figure 1:**
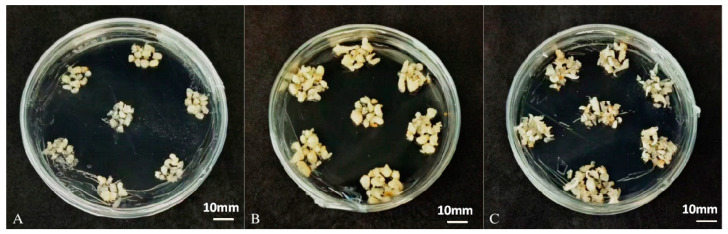
Morphological observation of *Areca catechu* L. embryogenic callus. (**A**) 0 day; (**B**) 30 days; (**C**) 60 days.

Morphological observation revealed that embryogenic callus initially appeared pale yellow-green and translucent (Figure 1A). After 30 days of culture, the callus gradually turned milky white (Figure 1B), and by day 60, well-formed, compact somatic embryos were visible on the surface of the callus (Figure 1C). Scale bars: 2 mm for A–C, 500 μm for D. All panels represent Treatment 7 (Y3 + 6-BA + TDZ + 30 g/L sucrose).These results demonstrate that the combination of 6-BA and TDZ synergistically promotes the acquisition of embryogenic competence and subsequent embryo differentiation in areca palm, whereas excessive exogenous auxinic herbicides are detrimental to embryogenic progression.

### 2.2. Effect of Basal Media on Somatic Embryo Differentiation

Using the optimal PGR combination (6-BA + TDZ), we compared six basal media with different nutrient profiles, including nitrogen content, NO_3_^−^/NH_4_^+^ ratios, N:P:K ratios, and trace element compositions. After 60 days of culture, significant differences in somatic embryo induction and browning mortality were observed among the media (Table 2). Y3 medium gave an induction rate of 11.33 ± 1.15% with the lowest browning mortality (9.52 ± 2.93%), representing the best balance between differentiation efficiency and explant survival. WPM medium produced a slightly higher induction rate (12.57 ± 5.16%) but with a substantially higher mortality rate (16.38 ± 3.31%). In contrast, MS and White media performed poorly: MS medium had a high mortality rate (23.61 ± 4.59%) despite a moderate induction rate (7.52 ± 1.83%), while White medium gave the lowest induction rate (2.38 ± 0.59%). Correlation analysis revealed that high ammonium content and a high N:P ratio (approximately 30:1:3 in MS) were associated with increased browning, whereas Y3 medium with a lower N:P ratio (approximately 5:1:2) and balanced trace elements provided a more favorable nutrient environment. These results indicate that areca embryogenic callus is sensitive to ammonium nitrogen, and that Y3 medium is the most suitable basal medium for somatic embryo differentiation.

### 2.3. Orthogonal Optimization of PGRs, Nitrogen Source, and Carbon Source

To further improve somatic embryo induction efficiency, a three-factor orthogonal experiment (L_12_ design) was conducted incorporating the PGR combination, basal medium (as a determinant of nitrogen source), and sucrose concentration. Twelve treatment combinations were tested, and the results are summarized in Table 3. Treatment 7—consisting of 6-BA + TDZ, the Y3 basal medium, and 30 g/L sucrose—achieved the highest somatic embryo induction rate (48.62 ± 4.83%), which was significantly higher than all other treatments. Moreover, this treatment maintained a low browning mortality rate (13.61 ± 0.71%). The induction rate obtained under this optimized condition was more than six times higher than that of the unoptimized control (7.76%) and nearly double that of the best single-factor treatment (Treatment 8 in the PGR screening, 27.40%).

Treatments with sucrose concentrations lower than 30 g/L (e.g., 10 or 20 g/L) generally resulted in lower induction rates, while higher sucrose concentrations (40 g/L) did not further improve induction and in some cases increased browning. These results suggest that 30 g/L sucrose provides an optimal balance between osmotic regulation and carbon/energy supply for embryogenic development. The synergistic effect among 6-BA + TDZ, Y3 medium, and 30 g/L sucrose indicates that the three factors collectively promote the reprogramming of somatic cells toward the embryogenic pathway and support the subsequent differentiation and maturation of somatic embryos.

### 2.4. Shoot Germination and Rooting

**Figure 2:**
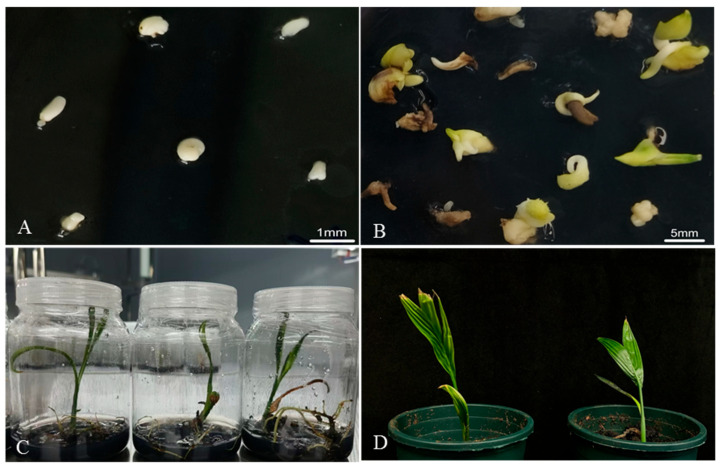
Areca embryoids regenerate into complete plants. (**A**) Embryoids; (**B**) Regenerated shoots; (**C**) Regenerated plantlets. (**D**) Seedlings after transplanting.

To optimize shoot germination from somatic embryos, different concentrations of 6-BA (1–4 mg/L) were tested using 1/2 MS basal medium (Figure 2A). After 30 days of light culture, the germination rate increased with 6-BA concentration up to 2 mg/L, then declined at higher concentrations (Table 4). The highest germination rate (65.63 ± 0.14%) was achieved at 2 mg/L 6-BA, accompanied by the lowest mortality rate (9.33 ± 0.08%). At 1 mg/L 6-BA, the germination rate was 47.46 ± 0.13%, while at 3 and 4 mg/L, germination rates decreased to 61.69 ± 0.06% and 55.53 ± 0.12%, respectively, with mortality rates increasing to >23%. The control treatment (no 6-BA) gave a low germination rate (28.33%) and high mortality (23.33%). These results indicate that 6-BA at an appropriate concentration (2 mg/L) promotes shoot differentiation from somatic embryos, whereas excessive cytokinin activity becomes inhibitory (Figure 2B).

Shoots with at least two leaves were transferred to the 1/2 MS medium without PGRs for root induction. Under light culture conditions, root primordia emerged within 15–20 days. For shoots exhibiting excessive taproot elongation without lateral roots, pruning of the taproot effectively stimulated lateral root formation. After 30–60 days, plantlets developed well-branched root systems and two true leaves (Figure 2C). Plantlets were successfully acclimatized and transplanted to a substrate of coconut coir and vermiculite (5:1, *v*/*v*). With standard greenhouse management (regular fertilization and pest control), transplanted plantlets grew normally without visible morphological abnormalities (Figure 2D). These results demonstrate that the optimized SE system supports complete plant regeneration from somatic embryos through to soil-acclimatized plants.

### 2.5. Cytological Characterization of Embryogenic and Non-Embryogenic Callus

**Figure 3:**
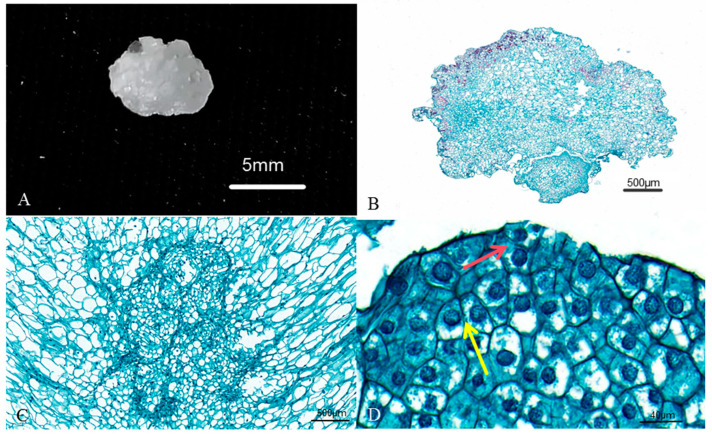
Cytological observation of *Areca catechu* embryonic callus. (**A**) Embryonal callus; (**B**) Embryonal callus section (10×); (**C**) Embryonal callus section (20×); (**D**) Localized embryonal callus section (40×). The red arrow indicates radial division, and the yellow arrow indicates pericyclic division.

To establish reliable criteria for selecting high-quality embryogenic callus and to understand the cellular basis of embryogenic competence, paraffin sectioning and cytological analysis were performed on embryogenic versus non-embryogenic callus. Embryogenic callus exhibited typical meristematic cytological features: cells were small, isodiametric or slightly oval, and densely packed with minimal intercellular spaces (Figure 3B,C). It is important to distinguish between “competent cells”—which are cells within the embryogenic callus that have acquired embryogenic potential but have not yet initiated embryo development—and “pro-embryos,” which are formed when a subset of competent cells undergo further epigenetic reprogramming to establish a defined embryonic morphology. The cytoplasm was dense, vacuolation was minimal, and the nucleus was large and centrally positioned, containing decondensed chromatin (chromatin in a loosely packed state that is permissive for transcription), reflecting high transcriptional activity. In contrast, although non-embryogenic cells also possessed large nuclei, their chromatin often appeared more condensed or irregularly organized. These features are characteristic of actively dividing cells with high embryogenic potential (Figure 3D). Quantitative measurements confirmed that embryogenic cells were significantly smaller than non-embryogenic cells in length (0.076 mm vs. 0.120 mm, *p* < 0.01), width (0.051 mm vs. 0.080 mm, *p* < 0.01), and area (0.0030 mm^2^ vs. 0.0079 mm^2^, *p* < 0.001) (Table 5).

**Figure 4:**
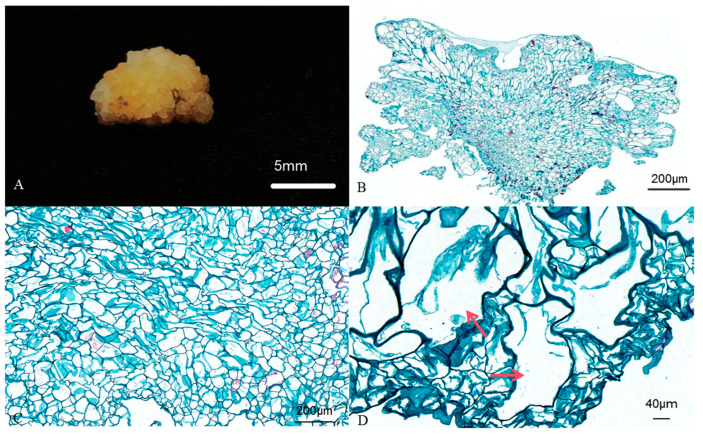
Cytological observation of non-embryonic callus of Areca catechu. (**A**) Non-embryonic callus; (**B**) Non-embryonic callus section (10×); (**C**) Non-embryonic callus tissue section (20×); (**D**) Local non-embryonic cell section (40×); the arrow indicates vacuolated cells.

In contrast, non-embryogenic callus consisted of large, irregularly shaped cells with sparse cytoplasm and extensive vacuolation (Figure 4B,C). The vacuoles often occupied most of the cell volume, compressing the nucleus to the periphery (Figure 4D). These cells lacked mitotic activity and showed no capacity for embryo differentiation. Two modes of embryogenic cell origin were observed in areca embryogenic callus. The first was external initiation, in which embryogenic cells arise from the callus surface via divisions oriented both parallel (periclinal-like) and perpendicular (anticlinal-like) to the surface (Figure 3D, red and yellow arrows, respectively). The second was internal initiation, in which the embryogenic cell clusters form deep within the callus tissue (Figure 3C). This dual origin indicates that embryogenic competence can be acquired by somatic cells in both peripheral and internal positions, consistent with observations in other palm species. From a developmental perspective, these cytological hallmarks—small cell size, dense cytoplasm, large nucleus with decondensed chromatin, and minimal vacuolation—represent a “competent” cellular state that is primed for embryogenic differentiation. Future studies investigating the epigenetic status of these embryogenic cells—including both DNA methylation patterns and histone modifications (particularly the histone methylation/acetylation ratio)— could provide early markers for predicting embryogenic competence before morphological changes become visible.

### 2.6. Histological Tracking of Somatic Embryo Development

**Figure 5:**
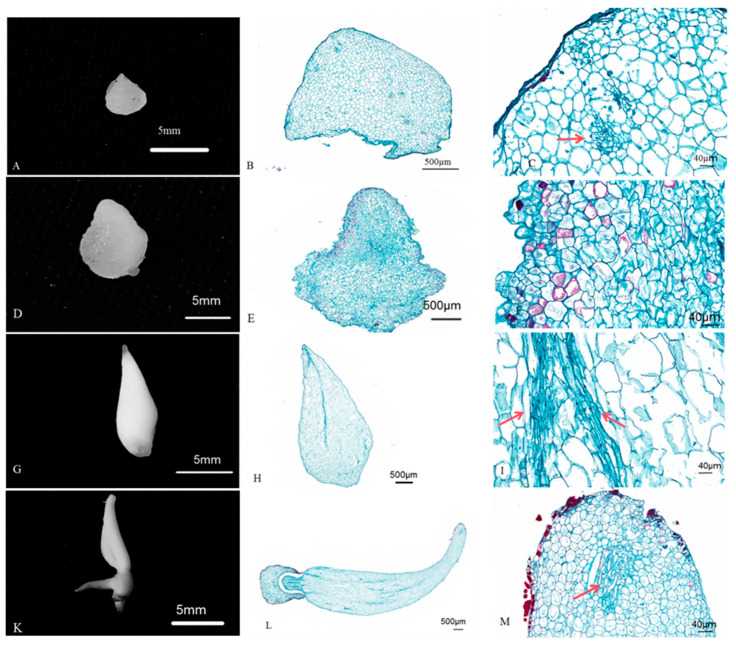
Histological observation of the different embryoids of Areca. (**A**): Spherical embryo; (**B**): Section (10×); (**C**): Localized section (40×), the arrow indicates the meristem end. (**D**) Shield-shaped embryo; (**E**) Section (10×); (**F**) Localized (40×). (**G**) Torpedo embryo; (**H**) Section (10×); (**I**): Localized section (40×), the arrow indicates the vascular bundle tissue. (**K**) somatic embryo; (**L**) Section (10×); (**M**) localized section (40×), the arrow indicates the shoot tip tissue.

To verify whether areca somatic embryos faithfully recapitulate the ontogenetic program of zygotic embryos, we examined paraffin sections of somatic embryos at four successive developmental stages: globular, shield-shaped, torpedo, and cotyledonary (mature) embryos. Globular stage: Somatic embryos at this stage appeared as spherical cell masses with densely packed cells (Figure 5A). The peripheral cells formed a protoderm-like layer, while internal cells were uniformly meristematic with no visible tissue differentiation (Figure 5B,C). This architecture mirrors that of early zygotic embryos and represents the initial establishment of embryo polarity. Shield-shaped stage: The embryo elongated along the apical–basal axis, and the first signs of tissue differentiation became apparent (Figure 5D). The apical domain exhibited smaller, actively dividing cells, whereas the basal domain began to show more elongated cell types (Figure 5E,F). This stage marks the transition from radial to bilateral symmetry and the initiation of organ primordia.

Torpedo stage: The embryo assumed an elongated torpedo shape (Figure 5G). Procambial strands became distinguishable as longitudinally oriented files of densely stained cells, indicating the onset of vascular differentiation (Figure 5H,I, arrows). The apical meristem and root primordium were more clearly delineated, establishing the bipolar structure characteristic of somatic embryos. Cotyledonary (mature) stage: The embryo reached its final size and shape, with a well-developed cotyledon and a distinct shoot apical meristem (Figure 5K,L, arrow). The procambium had further differentiated, and storage reserves accumulated in the cotyledonary cells (Figure 5M). The overall anatomy closely resembled that of mature zygotic embryos of areca palm. These histological observations demonstrate that somatic embryos of areca palm progress through a developmental sequence that is morphologically and structurally equivalent to that of zygotic embryos, confirming the authentic nature of the SE process and the successful activation of the complete embryogenic program in somatic cells under the optimized culture conditions.

## 3. Discussion

The acquisition of embryogenic competence is a complex process regulated by the interplay of endogenous hormone synthesis and gradient formation. Exogenous plant growth regulators (PGRs) act as chemical cues to modulate these internal pathways [17,18]. In this study, the combination of two cytokinins, 6-BA and TDZ, significantly outperformed treatments dominated by exogenous auxinic herbicides such as 2,4-D or Picloram in promoting somatic embryo differentiation in areca palm. Under the optimal 6-BA + TDZ regime, the induction rate reached 27.40% (further increased to 48.62% after orthogonal optimization), whereas treatments containing 2,4-D or Picloram resulted in high browning mortality (>22%) and low induction rates.

It has been proposed that TDZ, a potent cytokinin with high stability, can act as an inducer of endogenous auxin and gibberellin synthesis. Therefore, the effectiveness of the 6-BA + TDZ combination is likely due to its ability to promote the de novo synthesis and proper gradient formation of endogenous auxin, rather than providing an exogenous auxin source. In contrast, treatments dominated by exogenous auxinic herbicides such as 2,4-D and Picloram maintain callus proliferation but fail to support organized embryo development. High concentrations of these synthetic compounds can disrupt endogenous auxin gradient formation and induce the accumulation of stress-related metabolites, leading to oxidative stress, membrane damage, and ultimately browning and cell death [22]. The low browning mortality observed under 6-BA + TDZ (9.53%) compared to auxinic herbicide treatments (>22%) supports this interpretation. Thus, for areca SE, the key to successful embryo development is the use of PGRs that induce a proper endogenous auxin response, rather than the application of exogenous auxinic herbicides that disrupt it.

Browning is a major obstacle in palm tissue culture [9]. In this study, browning mortality varied from 9.52% (Y3) to 23.61% (MS). The key differentiating factor was not simply the absolute concentration of a single nutrient, but rather the overall nutrient balance. The MS medium, with its high ammonium content (288.8 mg/L) and a specific N:P:K ratio (approximately 30:1:3), caused the highest mortality. The Y3 medium, with moderate ammonium (52.5 mg/L), a lower N:P:K ratio (approximately 5:1:2), and a balanced trace element composition, gave the lowest mortality. The white medium (no NH_4_^+^) gave the lowest induction rate, indicating that some ammonium is necessary but excess is harmful. Ammonium toxicity, when it occurs, arises from pH gradient disruption (rather than simple pH change), depletion of energy reserves via the GS/GOGAT pathway, and ROS-induced oxidative damage Areca embryogenic callus, when cultured in darkness, relies entirely on heterotrophic metabolism and lacks photosynthetic capacity to efficiently assimilate excess ammonium. The Y3 medium provides a physiologically balanced nitrogen environment that supports embryogenic development while minimizing ammonium-induced stress, and 30 g/L sucrose further supports this balance: sucrose serves as an energy source and a carbon skeleton provider for cell wall and macromolecule synthesis, and the optimal concentration likely alleviates water stress that could otherwise exacerbate ammonium toxicity [23].

Cytological analysis revealed that areca embryogenic callus consists of small, isodiametric cells with dense cytoplasm, large centrally positioned nuclei with decondensed chromatin (reflecting high transcriptional activity), and minimal vacuolation—hallmarks of meristematic cells that reflect high metabolic activity, active transcription, and pluripotency. In contrast, non-embryogenic callus cells are large, irregular, and highly vacuolated, indicating terminal differentiation and loss of regenerative capacity. These cytological features are conserved across plant species [5,24], suggesting a universal cellular basis for embryogenic competence. Most importantly, histological tracking from globular to cotyledonary stages demonstrated that areca somatic embryos faithfully recapitulate the zygotic developmental program, including bipolarity establishment, procambium differentiation, and apical meristem formation. This faithful recapitulation is the ultimate validation of any SE system, confirming that somatic cells have been successfully reprogrammed to follow the correct developmental trajectory. Future studies employing chromatin analysis techniques (e.g., ATAC-seq, ChIP-seq) could provide deeper insights into the chromatin dynamics underlying the transition to embryogenic competence. The acquisition of embryogenic competence is fundamentally an epigenetic reprogramming process. Competent cells exhibit characteristic chromatin features (decondensed, transcriptionally active chromatin) that distinguish them from non-competent cells.

The superior performance of 6-BA + TDZ in areca SE contrasts with many palm SE systems where auxins (especially 2,4-D) are dominant for embryogenic callus induction [9]. Our finding that cytokinin-dominant regimes outperform auxin-dominant ones for differentiation aligns with the general principle that auxin promotes proliferation while cytokinin promotes differentiation [25]. However, in areca, even the induction phase appears to benefit from cytokinin dominance, suggesting species-specific differences in PGR sensitivity. The ammonium sensitivity we observed is consistent with reports in coconut, where embryogenic callus also performs better on Y3 than on MS medium [12]. It is worth noting that 1/2 MS medium, which has a high N:P ratio (approximately 48:1), was used for shoot germination and rooting rather than for embryo differentiation. This high N:P ratio, which promotes vegetative growth in many species, may be suboptimal for somatic embryo differentiation but could be suitable for the post-embryonic developmental stages, possibly due to species-specific mineral requirements. Together, these comparisons indicate that while some principles of SE are universal (e.g., the requirement for balanced nitrogen, the cytological features of embryogenic cells), optimal PGR regimes must be empirically determined for each species.

In summary, the optimized SE system described here significantly improves somatic embryo differentiation efficiency in areca palm through the synergistic action of 6-BA and TDZ, which likely act by inducing endogenous auxin synthesis. The use of the Y3 medium with its balanced nutrient profile minimizes browning and supports embryogenic development. Cytological evidence confirms that areca somatic embryos faithfully follow the zygotic developmental program. These findings provide a robust foundation for large-scale clonal propagation of elite areca germplasm and for future studies on the molecular mechanisms of somatic cell reprogramming in this tropical woody monocot.

## 4. Materials and Methods

### 4.1. Plant Materials and Culture Conditions

Embryogenic callus of areca palm (*Areca catechu* L.) previously induced from immature inflorescence explants following a previously established protocol [26]. Briefly, immature inflorescences (5–10 cm in length) were collected from adult areca palms, surface-sterilized with 70% ethanol for 1 min followed by 0.1% HgCl_2_ for 8 min, and rinsed three times with sterile distilled water. Explants were cultured on Y3 basal medium supplemented with 1.0 mg/L 2,4-D, 0.5 mg/L 2ip, 30 g/L sucrose, and 2.5 g/L phytagel, pH 5.8, in darkness at 26 ± 1 °C for 60 days to induce embryogenic callus. The callus was maintained on a proliferation medium consisting of Y3 basal medium supplemented with 1 mg/L 2,4-D and 0.5 mg/L 2ip and subcultured every 28 days. Unless otherwise specified, all cultures were incubated at 26 ± 1 °C in darkness with a relative humidity of 60%. For shoot germination and rooting, cultures were transferred to a photoperiod of 16 h light (LED, approximately 50–60 µmol·m^−2^·s^−1^, measured as Photosynthetic Photon Flux Density (PPFD) and 8 h darkness. Each treatment consisted of three independent replicates, and each replicate contained five culture vessels.

### 4.2. Effect of Plant Growth Regulator Combinations

The PGR concentrations and combinations tested in this study were selected based on previously published protocols for areca palm and other palm species [9,14], as well as preliminary single-factor experiments conducted in our laboratory. Concentrations were chosen within the following ranges commonly used in palm tissue culture: 2,4-D at 1–2 mg/L, 6-BA at 1–3 mg/L, TDZ at 0.5–1.5 mg/L, and auxins (NAA, IAA) at 0.5–1.0 mg/L. The nine treatment combinations were designed to evaluate the effects of different PGR types and their interactions on somatic embryo differentiation. To evaluate the effects of different PGR combinations on somatic embryo differentiation, nine treatment groups were designed based on the Y3 basal medium supplemented with various PGRs (Table 1). The unoptimized control consisted of 2.0 mg/L 2,4-D and 0.5 mg/L Kin in Y3 medium. For each treatment, 70 uniform spherical embryogenic calli (5–8 mm in diameter, pale yellow-green and translucent) were inoculated per Petri dish, with five dishes per replicate and three replicates per treatment. After 60 days of dark culture, the somatic embryo induction rate (percentage of callus pieces that produced at least one visible somatic embryo) and the browning mortality rate (percentage of callus pieces that turned completely brown and showed no signs of viability) were recorded.

### 4.3. Effect of Basal Media

Based on the optimal PGR combination identified above (6-BA + TDZ), six basal media—1/2 MS, Y3, MS, WPM, White, and B5—were tested for their effects on somatic embryo differentiation. The nitrogen content, NO_3_^−^/NH_4_^+^ ratio, N:P:K ratio, and trace element composition of each basal medium were compiled. Seventy embryogenic calli were inoculated per dish, with five dishes per replicate and three replicates per treatment. Induction and mortality rates were assessed after 60 days.

### 4.4. Orthogonal Optimization of PGRs, Nitrogen Source, and Carbon Source

A three-factor orthogonal experiment (L_12_ design) was conducted to optimize the combined effects of PGRs, the basal medium (as a determinant of nitrogen source), and sucrose concentrations (10, 20, 30, or 40 g/L) on somatic embryo differentiation. Each treatment was performed with three replicates as described above. After 60 days, the induction and mortality rates were evaluated to determine the optimal combination.

### 4.5. Somatic Embryo Germination and Rooting

Healthy somatic embryos obtained from the optimized differentiation protocol were transferred to germination medium consisting of 1/2 MS basal medium supplemented with different concentrations of 6-BA (1, 2, 3, or 4 mg/L). 1/2 MS medium without 6-BA served as the control. Twenty somatic embryos were inoculated per dish, with three replicates per treatment. After 30 days of culture under light conditions (16 h light/8 h dark, 2000–3000 lx, 28 ± 1 °C), the germination rate (percentage of embryos producing visible shoots) and mortality rate were recorded.

Shoots with at least two leaves were transferred to rooting medium (1/2 MS basal medium) without PGRs. The cultures were maintained under the same light conditions. For shoots exhibiting excessive taproot elongation without lateral roots, the taproot was pruned to stimulate lateral root formation. After 30–60 days, plantlets with well-developed root systems and two true leaves were acclimatized and transplanted to a substrate consisting of coconut coir and vermiculite (5:1, *v*/*v*). Transplanted plantlets were maintained under greenhouse conditions with regular fertilization and pest management.

### 4.6. Cytological Observation

Embryogenic and non-embryogenic calli, as well as somatic embryos at different developmental stages (globular, shield-shaped, torpedo, and cotyledonary stages), were fixed in FAA solution (50% ethanol:formalin:acetic acid = 90:5:5, *v*/*v*) for 24 h at 4 °C. After fixation, samples were dehydrated through a graded ethanol series (70%, 80%, 90%, 95%, and 100%), cleared in xylene, and embedded in paraffin wax. Sections of 8–10 μm thickness were cut using a rotary microtome (Leica RM2235, Leica Biosystems, Wetzlar, Germany), stained with safranin and fast green, and observed under a light microscope (Nikon Eclipse Ci-L, Japan). Cell length, width, and area were measured using NIS-Elements imaging software(Ver.6.20). For each callus type, at least five randomly selected fields of view (20× objective) were analyzed, and 20 cells per field were measured.

### 4.7. Statistical Analysis

All data were analyzed using IBM SPSS Statistics 26.0. One-way analysis of variance (ANOVA) followed by Duncan‘s multiple range test was performed to compare differences among treatments. Statistical significance was set at *p* < 0.05. Data are presented as mean ± standard error (SE). Graphs were generated using GraphPad Prism 9.0.

## Figures and Tables

**Table 1:** Effects of different PGR combinations on somatic embryo induction and browning mortality after 60 days of culture.

Treatment	PGR Combination (mg/L)	Induction Rate (%)	Browning Mortality (%)
1 (Control)	2,4-D 2.0 + Kin 0.5	7.76 ± 2.87	3.10 ± 1.10
2	6-BA 2.0 + 2ip 0.5 + NAA 0.5 + IAA 0.5	12.27 ± 2.64	7.14 ± 0.75
3	TDZ 1.0 + NAA 0.5	11.76 ± 2.78	8.47 ± 1.50
4	6-BA 2.0 + 2ip 0.5 + NAA 0.5 + IAA 0.5 + KN 0.5 + NOA 0.5	17.16 ± 3.03	9.73 ± 1.40
5	2ip 1.0 + NAA 0.5	21.07 ± 3.60	19.67 ± 2.57
6	2ip 1.0 + IAA 0.5	15.53 ± 4.61	24.57 ± 4.48
7	6-BA 2.0 + Pic 1.0	16.79 ± 6.22	25.33 ± 5.57
8	TDZ 1.0 + 6-BA 2.0	27.40 ± 3.60	9.53 ± 1.61
9	6-BA 2.0 + 2,4-D 1.0	16.07 ± 7.11	22.46 ± 1.41

Abbreviations: 6-BA, 6-benzyladenine; 2ip, 2-isopentenyladenine; NAA, α-naphthaleneacetic acid; IAA, indole-3-acetic acid; TDZ, thidiazuron; KN, kinetin; NOA, 2-naphthoxyacetic acid; Pic, picloram; 2,4-D, 2,4-dichlorophenoxyacetic acid.

**Table 2:** Effects of different basal media on somatic embryo induction and browning mortality after 60 days of culture.

Treatment	Basal Medium	Induction Rate (%)	Browning Mortality (%)
1	1/2 MS	3.71 ± 0.57	17.33 ± 5.25
2	Y3	11.33 ± 1.15	9.52 ± 2.93
3	MS	7.52 ± 1.83	23.61 ± 4.59
4	WPM	12.57 ± 5.16	16.38 ± 3.31
5	White	2.38 ± 0.59	14.85 ± 2.34
6	B5	8.09 ± 2.14	21.99 ± 3.86

Abbreviations: MS, Murashige and Skoog medium; Y3, Eeuwens‘ Y3 basal medium; 1/2 MS, half-strength Murashige and Skoog medium; WPM, Woody Plant Medium; White, White’s medium; B5, Gamborg‘s B5 medium.

**Table 3:** Orthogonal experimental design (L_12_) for optimization of PGR combination.

Treatment	PGR Combination (mg/L)	Basal Medium	Sucrose (g/L)	Induction Rate (%)	Browning Mortality (%)
1	2ip 1.0 + NAA 0.5	Y3	20	14.63 ± 0.86	16.08 ± 0.61
2	2ip 1.0 + NAA 0.5	WPM	10	17.31 ± 0.61	20.27 ± 1.24
3	2ip 1.0 + NAA 0.5	WPM	30	24.53 ± 1.81	18.09 ± 1.19
4	2ip 1.0 + NAA 0.5	B5	40	19.32 ± 0.65	22.37 ± 0.91
5	6-BA 2.0 + TDZ 1.0	B5	10	8.57 ± 0.77	23.66 ± 1.28
6	6-BA 2.0 + TDZ 1.0	B5	20	15.78 ± 1.06	13.79 ± 0.90
7	6-BA 2.0 + TDZ 1.0	Y3	30	48.62 ± 4.83	13.61 ± 0.71
8	6-BA 2.0 + TDZ 1.0	WPM	40	30.67 ± 2.30	10.85 ± 0.85
9	6-BA 2.0 + NAA 0.5 + NOA 0.5 + IAA 0.5 + 2ip 0.5 + KN 0.5	Y3	10	9.00 ± 1.00	17.86 ± 0.79
10	6-BA 2.0 + NAA 0.5 + NOA 0.5 + IAA 0.5 + 2ip 0.5 + KN 0.5	Y3	40	23.33 ± 0.57	21.19 ± 0.73
11	6-BA 2.0 + NAA 0.5 + NOA 0.5 + IAA 0.5 + 2ip 0.5 + KN 0.5	B5	30	26.00 ± 5.97	19.90 ± 1.19
12	6-BA 2.0 + NAA 0.5 + NOA 0.5 + IAA 0.5 + 2ip 0.5 + KN 0.5	WPM	20	23.34 ± 3.93	23.52 ± 1.40

Abbreviations: 6-BA, 6-benzyladenine; 2ip, 2-isopentenyladenine; NAA, α-naphthaleneacetic acid; IAA, indole-3-acetic acid; TDZ, thidiazuron; KN, kinetin; NOA, 2-naphthoxyacetic acid; Y3, Eeuwens‘ Y3 basal medium; WPM, Woody Plant Medium; B5, Gamborg’s B5 medium.

**Table 4:** The effects of 6-BA on Embryo Budding.

No	6-BA (mg/L)	Budding Rate (%)	Mortality Rate (%)
1	0	28.33 ± 0.13	23.33 ± 0.19
2	1	47.46 ± 0.13	17.67 ± 0.07
3	2	65.63 ± 0.14	9.33 ± 0.08
4	3	61.69 ± 0.06	23.00 ± 0.05
5	4	55.53 ± 0.12	26.33 ± 0.08

**Table 5:** Comparison of Embryonic Callus and Non-embryonic Callus of Areca Palm.

Type	Cell Length (mm)					
	1	2	3	4	5	Average
Embryonic	0.07896	0.08143	0.08945	0.06684	0.06405	0.07615
Non-Embryonic	0.12889	0.11880	0.11474	0.11997	0.11836	0.12015
Type	Cell width (mm)					
	1	2	3	4	5	Average
Embryonic	0.04577	0.04828	0.05590	0.05186	0.05326	0.05101
Non-Embryonic	0.07674	0.07094	0.09464	0.07939	0.07938	0.08022
Type	Cell area (mm^2^)					
	1	2	3	4	5	Average
Embryonic	0.00291	0.00278	0.00340	0.00298	0.00307	0.00303
Non-Embryonic	0.00749	0.00800	0.00830	0.00748	0.00802	0.00786

## Data Availability

Data will be made available on request.
